# Human Bocavirus, a Respiratory and Enteric Virus

**DOI:** 10.3201/eid1304.061501

**Published:** 2007-04

**Authors:** Diego Vicente, Gustavo Cilla, Milagrosa Montes, Eduardo G. Pérez-Yarza, Emilio Pérez-Trallero

**Affiliations:** * Hospital Donostia, San Sebastián, Spain; † Universidad del País Vasco, San Sebastián, Spain

**Keywords:** respiratory tract infection, gastroenteritis, parvovirus, bocavirus, dispatch

## Abstract

In Spain, human bocavirus (HBoV) was detected in 48 (9.1%) of 527 children with gastroenteritis at similar frequency as for children with respiratory illness (40/520, 7.7%). Fecal excretion adds new concern about the transmission of HBoV. To our knowledge, this report is the first to document HBoV in human feces.

Human bocavirus (HBoV), a recently described new virus species belonging to the *Parvoviridae* family, was identified as a human pathogen in September 2005 (*1*). Since then, this parvovirus has been found in children with respiratory tract illness in practically all areas of the world in which it has been investigated (*2*–*5*), an indication of its wide dissemination.

## The Study

To determine the prevalence and clinical characteristics of HBoV, we investigated the presence of this virus in children with respiratory tract infection in our region (Gipuzkoa, Basque country, Spain). Among the first patients in whom HBoV was detected in nasopharyngeal aspirates, we found two 12-month-old children with diarrhea in addition to respiratory symptoms. Because animal parvoviruses are frequently associated with enteritis in young animals (*6*), we investigated the presence of HBoV in the diarrheal feces of both children. HBoV was detected in both samples, and no other intestinal pathogens were identified. To rule out the possibility that this result could have been due to fecal contamination resulting from swallowing respiratory secretions, and to determine whether the gastrointestinal tract is affected by this new respiratory virus, we studied its presence in patient feces in 527 episodes of acute gastroenteritis, unrelated to respiratory infection, in children <3 years of age, mainly from nonhospital centers (ambulatory clinics). Our analysis were conducted from December 2005 through March 2006.

Viral DNA and RNA were obtained from nasopharyngeal aspirates and stool specimens with an automatic extractor BioRobot M48 (QIAGEN, Hilden, Germany) by using the MagAttract Virus Mini M48 kit (QIAGEN). cDNA was obtained by using M-MuLV reverse transcriptase (Promega, Madison, WI, USA) and random primers. Aliquots of the DNA and cDNA were frozen at −40°C until PCR for HBoV detection was performed. Respiratory samples were investigated for respiratory syncytial virus, influenza viruses A and B, parainfluenza virus types 1–4, and adenovirus by cell culture and PCR. Rhinovirus, coronavirus (NL63 coronavirus included), and metapneumovirus were studied by PCR alone. Fecal specimens were examined for *Shigella* spp., *Salmonella* spp., *Yersinia enterocolitica*, *Campylobacter* spp., and enteroinvasive *Escherichia coli* O157 by standard culture methods. Rotavirus was investigated by enzyme immunoassay and norovirus by reverse transcriptase PCR. HBoV detection was performed by PCR with primers derived from the *NP1* gene (*1*). Positive samples were retested and confirmed as positive by using a second PCR assay with primers derived from another location in the HBoV genome (*VP1* gene) (*7*). Amplified *NP1* and *VP1* gene fragments (354 bp and 403 bp, respectively) were sequenced and analyzed by using the BLAST software (www.ncbi.nlm.nih.gov/BLAST). Each PCR run included a negative control (water) that was treated as the clinical sample throughout, and PCR was performed with the usual precautions to avoid contamination. Strain *Spain001* (GenBank accession no. EF186830) was included as positive control in each PCR run.

## Conclusions

Of the 527 stool samples analyzed from December 2005 through March 2006, HBoV was detected in 48 (9.1%). From a second group of 520 children <3 years of age who came to the pediatric emergency unit of our hospital with an episode of acute respiratory infection during the same period, a similar frequency of HBoV detection was obtained (40/520, 7.7%) when nasopharyngeal aspirates were tested. Analysis of *NP1* and *VP1* partial gene sequences obtained from all fecal and respiratory HBoV-positive samples showed a similarity of >95% with previously published HBoV sequences.

Of 40 HBoV-positive respiratory samples, 25 (62.5%) showed coinfection with other viruses (respiratory syncytial virus in 13, rhinovirus in 3, influenza A in 3, coronavirus OC43 in 2, adenovirus in 1, influenza B in 1, respiratory syncytial virus and coronavirus OC43 in 1, and influenza A and rhinovirus in 1). Of the 48 HBoV-positive fecal samples, 28 (58.3%) showed coinfection with another intestinal pathogen (*Salmonella enteritidis* in 1, *Campylobacter jejuni* in 5, rotavirus in 14, norovirus in 7, and *C. jejuni* and norovirus in 1).

In this study, simultaneous detection of HBoV and other agents was frequent for respiratory or enteric specimens. The incidence of coinfection in respiratory illness was similar to that observed in studies that were not limited to specimens that had already tested negative for other microorganisms and in which a wide number of agents were investigated (*4*). Adenoviruses have been associated with infection of the colon and the gut and are a cause of severe gastroenteritis in nonindustrialized countries. In this study, coinfection of adenovirus and HBoV was detected in 1 respiratory specimen but these viruses together were not detected in any fecal sample.

HBoV and parvovirus B19 are the only 2 species of the *Parvoviridae* family that have been associated with disease in humans. To date, HBoV has only been detected in samples from the respiratory tract and has been associated with both upper and lower respiratory tract disease in infants and young children. The results of our study show that HBoV is also present in the gastrointestinal tract in children with gastroenteritis with or without symptoms of respiratory infection. The fecal excretion adds new concern about the transmission of HBoV.

To our knowledge, this report is the first to document HBoV in human feces. The high frequency of HBoV detection in the feces of children with gastroenteritis and the absence of any other intestinal pathogen suggest that this new virus species is an enteric, as well as a respiratory, pathogen. Further investigations to confirm this preliminary hypothesis and gain greater knowledge of the association between HBoV and enteric disease are required.
